# Frontiers in Antimicrobial Biomaterials

**DOI:** 10.3390/ijms23169377

**Published:** 2022-08-19

**Authors:** Helena P. Felgueiras

**Affiliations:** Centre for Textile Science and Technology (2C2T), University of Minho, Campus de Azurém, 4800-058 Guimarães, Portugal; helena.felgueiras@2c2t.uminho.pt

Biomaterials can be used as implantable devices or drug delivery platforms, which have significant impacts on the patient’s quality of life. Indeed, every year, a substantial number of new biomaterials and scaffolding systems are engineered and introduced in the biomedical field, with increased health benefits observed, as reported by Spalek et al. [1]. However, their long-term use can be threatened by the adhesion and proliferation of microorganisms, which can interact and form biofilms, or by the formation of fibrosis and consequent triggered cytotoxic responses. Pathogenic microorganisms may cause local infections and lead to implant failures. Additionally, they can hinder the delivery of therapeutic molecules by specialized carriers, rendering them ineffective. Many alternatives have been proposed over the years to prevent such events, including the use of antiseptics and antibiotics or the physical modification of the biomaterial surface, with the incorporation of biomolecules having developed into an area of interest. From specialized polymers and functional groups to silver and, more recently, antimicrobial peptides and natural extracts, different functionalization and modification techniques have been employed in this fight against pathogenic agents [1,2,3,4,5,6,7]. Marin et al., for instance, introduced a new generation of collagen-based biomaterials embedded with nanoclay for skin regeneration, demonstrating an improved antimicrobial potential. They reported that, depending on the nanoclay type used, both the cellular viability and antimicrobial activity (potentiated by gentamicin) could be controlled for a prolonged action over time [8]. This Special Issue aims at furthering our understanding of the antimicrobial actions of specialized biomaterials and introducing new surface modification strategies, original polymeric chemical structures, and new antimicrobial agent–material combinations, from which infection control or microbial eradication can be achieved.

In this collection of research, many important findings can be highlighted, namely the engineering and synthesis of novel antimicrobial agents. Fadaka et al. took a well-known nanomaterial, the silver nanoparticles, and modified its synthesis to improve its physical-chemical properties. They used gum arabic, sodium borohydride, and their combination as reducing agents and evaluated the particles’ antimicrobial and cytotoxic profiles. Gum arabic was deemed to be the most effective reducing agent in improving the bactericidal efficiency of the synthesized silver nanoparticles. However, the authors concluded that the nanoparticle toxicity could not be completely overcome, even by using a greener synthesis methodology, which is required in order to establish ranges of effectiveness for human safety [9]. Novel aminothiazoles with superior antiviral, antioxidant, and antibacterial activities were also synthesized by Minickaitė et al. They demonstrated that, by using substitutes in the thiazole ring, optimized antimicrobial structures could be generated with target specificity [10]. Yussof et al. revealed similar outcomes when exploring the antibacterial and sporicidal effectiveness of theaflavin-3,3′-digallate. They determined the potential of this polyphenol, derived from the leaves of *Camellia sinensis*, to fight against a range of bacteria, including the spore-forming *Bacillus* spp., and established its promising broad-spectrum antibacterial and anti-spore activities [11].

Antimicrobial peptides are considered a new generation of antimicrobial agents. Indeed, in recent years, they have been explored as potential alternatives to antibiotics and other immunomodulatory drugs [12]. Umehara et al. studied the well-characterized skin-derived human β-defensins antimicrobial peptides and demonstrated their influence on the secretion of angiogenin, a potent angiogenic factor. They revealed that various human β-defensins can stimulate the production of angiogenin while maintaining their antimicrobial activities and other immunomodulatory properties [13]. Zhu et al. identified a new antimicrobial peptide gene, the Sparanegtin gene from the mud crab *Scylla paramamosain*, whose transcripts were particularly abundant in the testis of that species. The recombinant Sparanegtin was found to be effective against both Gram-positive and Gram-negative bacteria, including *Pseudomonas aeruginosa*, and its immunomodulatory effects on specific bacteria were also revealed [14]. Yang et al. also identified two male-specific antimicrobial peptides, SCY2 and Scyreprocin from the mud crab *Scylla paramamosain*, and established their dual role in reproductive immunity and sperm acrosome reactions while maintaining their antimicrobial profiles [15].

In light of the size and sensitivity to physiological conditions of most bioactive agents, including antimicrobial peptides, optimizing their localized and target deliveries are essential. With this in mind, Yang et al. proposed the incorporation of ClyF, an anti-staphylococcal lysin, into constructs of silica-binding peptide for application as device coatings for the prevention of *Staphylococcal*-related infections. The ClyF-immobilized surfaces supported the normal attachment and growth of mammalian cells and displayed significant bactericidal features, being deemed potentially effective in preventing the growth of antibiotic-resistant microorganisms [16]. In turn, Egle et al. studied the influence of an engineered platelet-rich fibrin, used as a carrier matrix in the antibacterial properties of clindamycin phosphate, on Gram-positive bacteria. The carrier was observed to induce structural changes in the clindamycin, giving rise to a more active compound that significantly decreased the minimal bactericidal concentrations required to eliminate *Staphylococcal* strains. The researchers attested to the safety of the engineered carrier for human cells in vitro, thus evidencing the system’s potential to reduce the risk of postoperative infection [17]. Finally, Lee et al. proposed the controlled storage and release of nitric oxide from metal organic nanosized frameworks formed from Cu-BTC for prospective uses in drug delivery systems. The nitric oxide release was maintained as constant for 12 h, meeting the requirements for clinical applications. Most importantly, the authors verified the structures’ antibacterial potential by their significant elimination of six bacteria strains, highlighting the synergistic effects between the payload and carrier [18].

## References

[1] Spałek J., Ociepa P., Deptuła P., Piktel E., Daniluk T., Król G., Góźdź S., Bucki R., Okła S. (2022). Biocompatible Materials in Otorhinolaryngology and Their Antibacterial Properties. Int. J. Mol. Sci..

[2] Teixeira M.A., Antunes J.C., Seabra C.L., Tohidi S.D., Reis S., Amorim M.T.P., Felgueiras H.P. (2022). Tiger 17 and pexiganan as antimicrobial and hemostatic boosters of cellulose acetate-containing poly (vinyl alcohol) electrospun mats for potential wound care purposes. Int. J. Biol. Macromol..

[3] Felgueiras H.P. (2021). An insight into biomolecules for the treatment of skin infectious diseases. Pharmaceutics.

[4] Antunes J.C., Domingues J.M., Miranda C.S., Silva A.F.G., Homem N.C., Amorim M.T.P., Felgueiras H.P. (2021). Bioactivity of chitosan-based particles loaded with plant-derived extracts for biomedical applications: Emphasis on antimicrobial fiber-based systems. Mar. Drugs.

[5] Felgueiras H.P., Homem N.C., Teixeira M., Ribeiro A., Teixeira M.O., Antunes J., Amorim M. (2021). Biodegradable wet-spun fibers modified with antimicrobial agents for potential applications in biomedical engineering. J. Phys. Conf. Ser..

[6] Teixeira M.O., Antunes J.C., Felgueiras H.P. (2021). Recent advances in fiber–hydrogel composites for wound healing and drug delivery systems. Antibiotics.

[7] Miranda C.S., Ribeiro A.R., Homem N.C., Felgueiras H.P. (2020). Spun biotextiles in tissue engineering and biomolecules delivery systems. Antibiotics.

[8] Marin M.M., Ianchis R., Leu Alexa R., Gifu I.C., Kaya M.G.A., Savu D.I., Popescu R.C., Alexandrescu E., Ninciuleanu C.M., Preda S. (2022). Development of New Collagen/Clay Composite Biomaterials. Int. J. Mol. Sci..

[9] Fadaka A.O., Meyer S., Ahmed O., Geerts G., Madiehe M.A., Meyer M., Sibuyi N.R.S. (2022). Broad Spectrum Anti-Bacterial Activity and Non-Selective Toxicity of Gum Arabic Silver Nanoparticles. Int. J. Mol. Sci..

[10] Minickaitė R., Grybaitė B., Vaickelionienė R., Kavaliauskas P., Petraitis V., Petraitienė R., Tumosienė I., Jonuškienė I., Mickevičius V. (2022). Synthesis of Novel Aminothiazole Derivatives as Promising Antiviral, Antioxidant and Antibacterial Candidates. Int. J. Mol. Sci..

[11] Yussof A., Cammalleri B., Fayemiwo O., Lopez S., Chu T. (2022). Antibacterial and Sporicidal Activity Evaluation of Theaflavin-3,3′-digallate. Int. J. Mol. Sci..

[12] Felgueiras H.P., Amorim M.T.P. (2017). Functionalization of electrospun polymeric wound dressings with antimicrobial peptides. Colloids Surf. B Biointerfaces.

[13] Umehara Y., Takahashi M., Yue H., Trujillo-Paez J.V., Peng G., Nguyen H.L.T., Okumura K., Ogawa H., Niyonsaba F. (2022). The Antimicrobial Peptides Human β-Defensins Induce the Secretion of Angiogenin in Human Dermal Fibroblasts. Int. J. Mol. Sci..

[14] Zhu X., Chen F., Li S., Peng H., Wang K.-J. (2022). A Novel Antimicrobial Peptide Sparanegtin Identified in Scylla paramamosain Showing Antimicrobial Activity and Immunoprotective Role In Vitro and Vivo. Int. J. Mol. Sci..

[15] Yang Y., Chen F., Qiao K., Zhang H., Chen H.-Y., Wang K.-J. (2022). Two Male-Specific Antimicrobial Peptides SCY2 and Scyreprocin as Crucial Molecules Participated in the Sperm Acrosome Reaction of Mud Crab Scylla paramamosain. Int. J. Mol. Sci..

[16] Yang W., Gondil V.S., Luo D., He J., Wei H., Yang H. (2021). Optimized Silica-Binding Peptide-Mediated Delivery of Bactericidal Lysin Efficiently Prevents Staphylococcus aureus from Adhering to Device Surfaces. Int. J. Mol. Sci..

[17] Egle K., Skadins I., Grava A., Micko L., Dubniks V., Salma I., Dubnika A. (2022). Injectable Platelet-Rich Fibrin as a Drug Carrier Increases the Antibacterial Susceptibility of Antibiotic—Clindamycin Phosphate. Int. J. Mol. Sci..

[18] Lee D.N., Kim Y.R., Yang S., Tran N.M., Park B.J., Lee S.J., Kim Y., Yoo H., Kim S.-J., Shin J.H. (2022). Controllable Nitric Oxide Storage and Release in Cu-BTC: Crystallographic Insights and Bioactivity. Int. J. Mol. Sci..

